# Distinct bilateral prefrontal activity patterns associated with the qualitative aspect of working memory characterized by individual sensory modality dominance

**DOI:** 10.1371/journal.pone.0238235

**Published:** 2020-08-26

**Authors:** Mayuko Matsumoto, Takeshi Sakurada, Shin-ichiroh Yamamoto

**Affiliations:** 1 College of Systems Engineering and Science, Shibaura Institute of Technology, Saitama, Japan; 2 Functional Brain Science Laboratory, Center for Development of Advanced Medical Technology, Jichi Medical University, Tochigi, Japan; 3 College of Science and Engineering, Ritsumeikan University, Shiga, Japan; 4 Department of Neurosurgery, Jichi Medical University, Tochigi, Japan; University of Pittsburgh, UNITED STATES

## Abstract

In addition to quantitative individual differences in working memory (WM) capacity, qualitative aspects, such as enhanced sensory modality (modality dominance), can characterize individual WM ability. This study aimed to examine the neurological basis underlying the individual modality dominance component of WM using functional near-infrared spectroscopy (fNIRS). To quantify the degree of individual WM modality dominance, 24 participants were required to find seven hidden targets and hold their spatial location and appearance order with vibrotactile or visual stimuli aids. In this searching task, eight participants demonstrated higher performance with the tactile condition (tactile-dominant) whereas sixteen demonstrated visual dominance. We then measured prefrontal activity by fNIRS during memorization of visual stimulus numbers while finger tapping as a cognitive-motor dual-task. Individual modality dominance significantly correlated with bilateral frontopolar and dorsolateral prefrontal activity changes over repeated fNIRS sessions. In particular, individuals with stronger visual dominance showed marked decreases in prefrontal area activity. These results suggest that distinct processing patterns in the prefrontal cortex reflect an individual’s qualitative WM characteristics. Considering the individual modality dominance underlying the prefrontal areas could enhance cognitive or motor performance, possibly by optimizing cognitive resources.

## Introduction

Functional working memory (WM) performance is one factor that represents an individual’s cognitive processing characteristics. WM refers to a cognitive system that holds and manipulates information for a given task over a short period of time [1] and relates to spatial information processing [2]. Several previous studies have focused on individual differences in quantitative WM aspects, such as memory capacity. Indeed, individual WM capacity predicts performance on a variety of cognitive [3, 4] and motor [5, 6] tasks. Evidence that individuals with greater abilities in specific domains, such as motor imagery vividness [7] and attentional control ability [8], also shows that superior motor learning indicates the importance of quantitative aspects of cognitive function.

In addition to the quantitative level, qualitative aspects influence individual cognitive function. We recently found that cognitive processing is dependent on efficient individual sensory processing (modality dominance) between internal body information, such as tactile or somatosensory stimuli, and external body information, such as visual stimuli [9–11]. These previous studies found that individuals with visual imagery dominance demonstrated better motor performance when using an external focus attentional strategy that required one’s attention toward a body movement outcome. Conversely, those with kinesthetic imagery dominance demonstrated better motor performance when using an internal focus attentional strategy that required one’s attention toward a body movement itself. It is currently unknown whether modality dominance is a qualitative WM parameter, like motor imagery and attentional strategy. However, considering sensory modality-dependence in WM, such as tactile and visual WM [12, 13], and the strong interactions between different cognitive functions, like attention, and WM [4, 14], we can expect that modality dominance is also a qualitative characteristic of individual WM abilities.

The neurological basis underlying qualitative individual differences in WM mediated by modality dominance is also unclear. Combined behavioral and neuroimaging studies have the potential to expand our knowledge of the neurological basis of modality dominance in WM. The bilateral prefrontal area in particular, including the dorsolateral prefrontal cortex (DLPFC) and frontopolar cortex (FPC), is widely recognized as a critical structure for WM. Previous studies have reported that the right DLPFC can maintain several forms of information such as visuospatial or tactile. In a transcranial direct current stimulation study, anodal stimulation over the right DLPFC improved accuracy for memorizing visuospatial locations [15]. In addition to the DLPFC, the FPC is also associated with visual spatial memory [16, 17]. While a study reported that the right prefrontal area contributes to the maintenance of tactile stimuli in WM [18], the majority have concluded that the right area mainly processes visuospatial information. By contrast, activation of the left DLPFC correlated with discrimination accuracy for two successive somatosensory stimuli, suggesting that the left DLPFC has an important role in WM for tactile information [19]. Left FPC activation was also associated with WM required to maintain representations of haptic information and to integrate spatial and motor components [20]. Furthermore, according to a meta-analysis, the bilateral DLPFC including Brodmann areas 9 and 46 show robust activity during n-back tasks requiring the monitoring and manipulation of spatial information [17]. Thus, the bilateral DLPFC and FPC are potential areas reflecting individual modality dominance in WM.

This pilot study was designed to examine whether modality dominance in WM varies among individuals and whether the prefrontal cortex is a neurological locus that reflects individual modality dominance. To evaluate individual WM modality dominance, healthy individuals performed a searching task that required them to hold spatially sequential patterns of vibrotactile or visual stimuli. We then used functional near-infrared spectroscopy (fNIRS) to observe the role of prefrontal neural activity in individual modality dominance. We predicted that different FPC or DLPFC activity would be observed according to the individual’s WM modality dominance evaluated in the first searching task. Specifically, our previous study exploring individual differences in attentional strategy [21] demonstrated that visual dominance was associated with less prefrontal cortex activity than tactile dominance. Therefore, if modality dominance widely influences cognitive functions, less prefrontal cortex activity should be observed in individuals that excel at holding visual information.

## Materials and methods

### Participants

Twenty-four healthy participants (mean age ± SD, 23.3 ± 4.6 years; 12 females) were recruited from the students at Jichi Medical University. All participants were right-handed as assessed by the Edinburgh Handedness Inventory (laterality 97.4 ± 7.70) [22]. This study was conducted in accordance with the Declaration of Helsinki and approved by the Institutional Review Board at Jichi Medical University. All participants provided written informed consent prior to participation. Each participant completed the following two tasks on the same day.

### Task 1: Quantifying individual WM modality dominance

The first task aimed to quantify the degree of WM modality dominance by evaluating performance in a searching task.

#### Experimental setup

**Fig 1:**
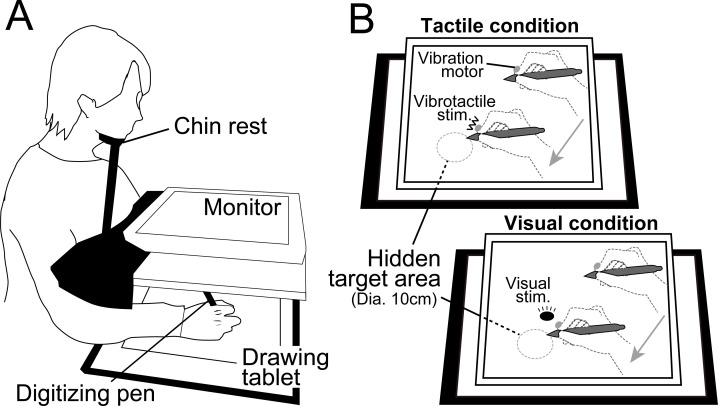
Experimental setup. (A) Each participant was required to find 7 hidden targets on a drawing tablet. (B) During the tactile condition, vibrotactile stimuli were used to inform target locations, and no information was presented on the monitor. Under the visual condition, a circular visual cursor (diameter: 5 cm) was presented indicating target locations.

Each participant was seated on a chair and asked to hold a digitizing-pen on a drawing tablet (Intuos4 PTK-1240/K0, Wacom, Japan) with their right hand. An LCD monitor (size: 30.5 cm × 37.7 cm) for visual stimulus presentation was placed horizontally 16.5 cm above the tablet at approximately 30 cm from the participants’ eyes such that they could not see their right hand directly during experimental tasks. Further, hand position was occluded using a cloth and by maintaining head position with a fixed chin rest (Fig 1A). Visual stimuli presented on the monitor were programmed in MATLAB (MathWorks, Natick, MA) using Cogent Toolbox software (University College London, London, UK, http://www.vislab.ucl.ac.uk/cogent.php). The position of the digitizing-pen tip was recorded using the Cogent Toolbox with sampling at 60 Hz. A vibration motor presenting vibrotactile stimuli was attached to the index fingertip of the right hand.

#### Procedure

The participants performed a searching task in which they were required to find 7 targets on the drawing tablet. These targets were randomly located and appeared sequentially one-by-one in a predetermined order. The search area on the drawing tablet was 30.5 cm × 37.7 cm and located directly below the monitor. First, participants moved the digitizing-pen to the center of the searching area. Then, after the background color of the monitor was changed as a start cue, the participants started to search for the first target. Participants were ultimately required to find all 7 targets as quickly as possible, so they had to hold spatial information, especially the target locations and appearance orders, in repeated trials.

For the searching task, we introduced two experimental conditions, a tactile condition and a visual condition that differed in cues used to hold the target locations and appearance orders. Target locations differed between tactile and visual conditions so there was no transfer across tasks. Under the tactile condition, when the tip of the digitizing-pen came into a target area on the tablet (diameter: 10 cm), a vibrotactile stimulus was presented on their right index finger from the vibration motor. By contrast, under the visual condition, a circular visual cursor was presented on the monitor just above the corresponding digitizing-pen position to indicate target location (Fig 1B). The vibrotactile or visual stimulus was presented continuously when the tip of the digitizing-pen was in a target area. Participants were instructed to maintain the position of the digitizing-pen for at least 0.7 s after finding a target. A beep signal was presented to inform the participant of successful target detection. Then the participants immediately started to search for the next target. A trial finished when the participant found all 7 targets.

The participants alternately performed 15 trials under each condition for 30 trials in total. The 7 target locations were consistent throughout all trials under each condition but differed between conditions. The first trial was randomly assigned as tactile or visual for each participant. To equalize task difficulty, the total distance among all targets (i.e., the sum of straight line distances connecting all targets from 1st to 7th) was the same for both conditions.

#### Analysis

To evaluate individual motor performance, we calculated searching time (Time) and normalized movement distance (Dis) in each trial. The searching time is the duration taken to find all targets, and the normalized movement distance is the movement distance of the right hand divided by the shortest distance connecting the 7 targets by a straight line. We defined the “searching cost” by the Eq (1)

$${SearchingCost}_{i}=\sqrt{{Time}_{i}^{2}+{\left({Dis}_{i}-1\right)}^{2}},$$ (1)

where subscript *i* denotes the trial number (1–15 in each condition). The searching cost in each trial indicates the distance from the (0, 1) coordinate on a Time–Dis plane. This index relates to individual WM ability because holding spatial information for hidden targets can contribute to optimizing the movement path. In other words, participants with an optimized path could move their hand to the next target in the shortest distance, thereby reducing the searching time. In this scenario, faster-reduced searching costs indicate a higher WM ability to efficiently hold target locations and appearance order.

To characterize the individual modality dominance in WM, we then compared average searching costs from 2nd to 15th trials under tactile and visual conditions. The first trial was eliminated because the participants could not know the target locations and so search cost would not reflect WM ability. We subtracted the average searching cost under the tactile condition from that under the visual condition as an index of modality dominance. Therefore, positive and negative values indicate tactile-dominant individual and visual-dominant individuals, respectively.

### Task 2: Recording prefrontal cortex using fNIRS

The second fNIRS task aimed to examine whether the prefrontal cortex is involved in qualitative individual WM differences. For this objective, we investigated whether prefrontal cortex activity depended on the modality dominance quantified in the first searching task.

#### Experimental setup

Each participant was seated on a chair facing an LCD monitor with their right hand holding a computer mouse on a desk. During the fNIRS task, the right hand was hidden by a small rack. For measurement of prefrontal activity, we used a multichannel fNIRS system (ETG-7100, Hitachi Medical Corporation, Kashiwa, Japan) with sampling at 10 Hz. The fNIRS probes were arranged to cover the prefrontal area. We used a 3 × 5 multichannel probe holder consisting of eight laser sources that emitted at 695 and 830 nm that were alternately arranged with seven detecting probes at an interprobe distance of 3 cm. The midpoint of an emitter/detector pair was defined as a recording channel location. The probe holder was placed on the scalp with its lowest-row center emitter at the participant’s Fpz position, according to the standard international 10–20 system (the fNIRS probes and holder setups were identical with our previous study [21]).

fNIRS signals reflect hemoglobin changes that originate in cortical tissue due to brain activation and skin blood flow. To eliminate skin blood flow’s impact on fNIRS signals, we applied multi-distance independent component analysis (ICA) [23–27]. The number of available recording channels was 15 after applying multi-distance ICA. To spatially register fNIRS maps onto the Montreal Neurological Institute coordinate space, we individually measured scalp landmarks and all fNIRS recording channel positions using a 3D magnetic space digitizer (FASTRAK, Polhemus, USA). We then used the position data from all participants’ recording channels to estimate spatial profiling without MRI [28]. The spatial profile of the recording channels is shown in Table 1.

**Table 1:** Spatial profiling of each recording channel.

CH	Localization	Broadman area	Probability
1	Left frontopolar cortex	10	1
2	Right frontopolar cortex	10	1
3	Left frontopolar cortex	10	0.972
	Left dorsolateral prefrontal cortex	46	0.028
4	Left frontopolar cortex	10	1
5	Right frontopolar cortex	10	1
6	Right frontopolar cortex	10	0.972
	Right dorsolateral prefrontal cortex	46	0.028
7	Left dorsolateral prefrontal cortex	46	0.496
	Left frontopolar cortex	10	0.349
	Left dorsolateral prefrontal cortex	9	0.105
	Left pars triangularis Broca's area	45	0.047
	Left inferior prefrontal gyrus	47	0.003
8	Left frontopolar cortex	10	0.902
	Left dorsolateral prefrontal cortex	9	0.098
9	Right frontopolar cortex	10	0.935
	Right dorsolateral prefrontal cortex	9	0.065
10	Right frontopolar cortex	10	0.888
	Right dorsolateral prefrontal cortex	9	0.112
11	Right dorsolateral prefrontal cortex	46	0.702
	Right frontopolar cortex	10	0.298
12	Left dorsolateral prefrontal cortex	9	0.628
	Left frontopolar cortex	10	0.186
	Left dorsolateral prefrontal cortex	46	0.145
	Left includes frontal eye fields	8	0.041
13	Left dorsolateral prefrontal cortex	9	0.770
	Left frontopolar cortex	10	0.127
	Left includes frontal eye fields	8	0.103
14	Right dorsolateral prefrontal cortex	9	0.741
	Right frontopolar cortex	10	0.171
	Right includes frontal eye fields	8	0.088
15	Right dorsolateral prefrontal cortex	9	0.657
	Right frontopolar cortex	10	0.199
	Right dorsolateral prefrontal cortex	46	0.144

#### Procedure

The fNIRS task consisted of eight sessions, each comprising seven alternating 15-s rest and task blocks with an additional rest block inserted at the end of each session (i.e., 15 blocks per session). During the task blocks, the participants were instructed to push the computer mouse button at about 1 Hz by tapping their finger. We additionally instructed individuals to count visual stimuli as the cognitive task. The monitor presented a red circular visual cursor with a random number of times in each task block. It should be noted that the effect of cognitive processing is not expected when a task is too easy for participants [29–31]. Further, individual differences in cognitive level may not appear when task difficulty is insufficient for participants [10]. Therefore, we applied a cognitive-motor dual-task for the task blocks to adequately increase task difficulty so that individual differences in WM ability could be easily observed in prefrontal cortex activity. We utilized a simple motion for the motor task to minimize motion artifacts in the fNIRS signals. By contrast, during the rest blocks, the participants were required to gesture the numbers of visual stimuli in the immediately preceding task block using their fingers. Except for the gesture, the participants did not need to perform any cognitive or motor task during the rest blocks.

#### Analysis

*Tapping frequency*. We calculated the mean tapping frequency for each session and subtracted the mean tapping frequency of the first two sessions from the mean of the last two sessions to evaluate changes in motor intensity during repeated fNIRS sessions (Δ*Freq*).

*Counting accuracy*. We calculated the counting accuracy for visual stimuli by dividing the number of blocks with a correct response by the total number of task blocks and excluded participants with accuracy of less than 90%.

*Preprocessing for fNIRS data*. To estimate local neural activity, we measured oxygenated hemoglobin (oxy-Hb) and deoxygenated hemoglobin (deoxy-Hb) signals. Individual oxy-Hb and deoxy-Hb signals of each channel were fitted to a first-degree polynomial. We then applied low-pass filtering at a cut-off frequency of 0.9 Hz to remove heartbeat pulsations. We expected task-related signals to be oxy-Hb and deoxy-Hb slow waves with a cycle close to the length of one block set (task block + rest block = 30 s). To remove waves that were much slower than the block cycle, we applied high-pass filtering at a cut-off frequency of 0.0167 Hz [1/(30 s × 2)]. Blocks with marked motion-related artifacts were removed. We referred to the Platform for Optical Topography Analysis Tools (POTATo; Research & Development Group, Hitachi, Ltd.) for these preprocesses.

Raw fNIRS signals are relative values and so cannot be directly compared or averaged across channels or participants. Thus, for comparison and statistical analysis, we first converted the preprocessed oxy-Hb and deoxy-Hb signals into z scores using the mean value and standard deviation of oxy-Hb and deoxy-Hb signal changes during the 10 s before starting each task block (baseline) [32]. We then averaged z time course data across available blocks in the 1st and 2nd sessions and the 7th and 8th sessions.

*Prefrontal activity*. As representative values of prefrontal activity level, we calculated the average z scores in the latter half of individual z time courses (i.e., from 7.5 to 15 s). We defined the average z score from the mean z profile across the 1st and 2nd sessions as the initial prefrontal activity (*Z*_*Init*_) and that from the mean z profile across the 7th and 8th sessions as the final prefrontal activity (*Z*_*Final*_). Further, to quantify the change of neural activity, we subtracted *Z*_*Init*_ from *Z*_*Final*_ (Δ*Z*).

### Statistical analysis

For the first searching task, average searching costs under the tactile and visual conditions were compared using a paired *t*-test. For the second fNIRS task, we calculated the Pearson correlation coefficient (r) between the inter-subject variance of modality dominance in WM (quantified in the first searching task) and that of prefrontal activities in the second fNIRS task (*Z*_*Init*_, *Z*_*Final*_, and Δ*Z*). A *p* < 0.05 (two-tailed) was considered significant for all tests.

## Results

### Task 1: Quantifying individual WM modality dominance

#### Searching cost and modality dominance in WM

No participants reported fatigue during the searching task. It took 18 ± 4.99 min to complete the 30 trials without any break.

**Fig 2:**
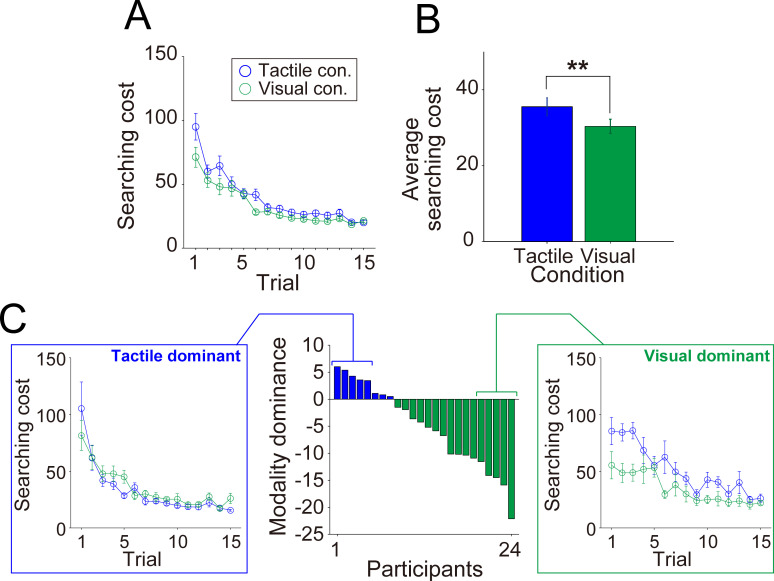
Searching cost transitions and individual WM modality dominance. The top panels show motor performance within the entire cohort, prior to individual modality dominance labeling. (A) Average trial-by-trial searching cost transition under tactile (blue) and visual (green) conditions. (B) Average searching cost under both conditions. (C) Differential searching costs between tactile and visual conditions sorted in descending order. Positive and negative values indicate individuals with tactile dominance (TD; blue bars) and visual dominance (VD; green bars), respectively. The left and right panels show the typical trial-by-trial searching cost transition in TD (participant nos. 1–5) and VD (participant nos. 20–24) individuals. Error bars denote the standard error. **p < 0.01.

Before labeling the individual modality dominance, we first compared motor performance between the tactile and visual conditions for the entire cohort. In both tactile and visual conditions, the participants gradually reduced searching cost over successive trials (Fig 2A). On average, the participants showed significantly lower searching cost under the visual condition compared to the tactile condition (Fig 2B [*p* = 0.0030, paired *t*-test]). However, modality dominance varied widely among participants, with eight participants showing lower searching costs under the tactile condition and sixteen under the visual condition. Based on differences in average searching cost between the tactile and visual conditions, we labeled the 24 participants as Tactile-dominant (TD) or Visual-dominant (VD) individuals (Fig 2C). The left and right panels of Fig 2C illustrate typical searching cost transitions in individuals with relatively stronger TD (participant nos. 1–5) and VD (participant nos. 20–24).

### Task 2: Recording prefrontal cortex using fNIRS

#### Tapping frequency

We confirmed that the participants performed continuous tapping movements with a slight increase in frequency (first two sessions: 1.28 ± 0.19 Hz, last two sessions: 1.43 ± 0.20 Hz; paired *t*-test, *p* = 0.061; Δ*Freq* range: −0.21 to 0.59 Hz).

#### Counting accuracy

As all participants showed counting accuracy over 90% (96.80 ± 0.49%), we analyzed all participants’ data in the second fNIRS task.

#### Prefrontal activity

The numbers of the blocks remaining for averaging z time courses after elimination of those with marked motion-related artifacts were 13.29 ± 0.27 blocks (94.94%) in the 1st and 2nd sessions and 13.13 ± 0.29 blocks (93.75%) in the 7th and 8th sessions, respectively.

**Fig 3:**
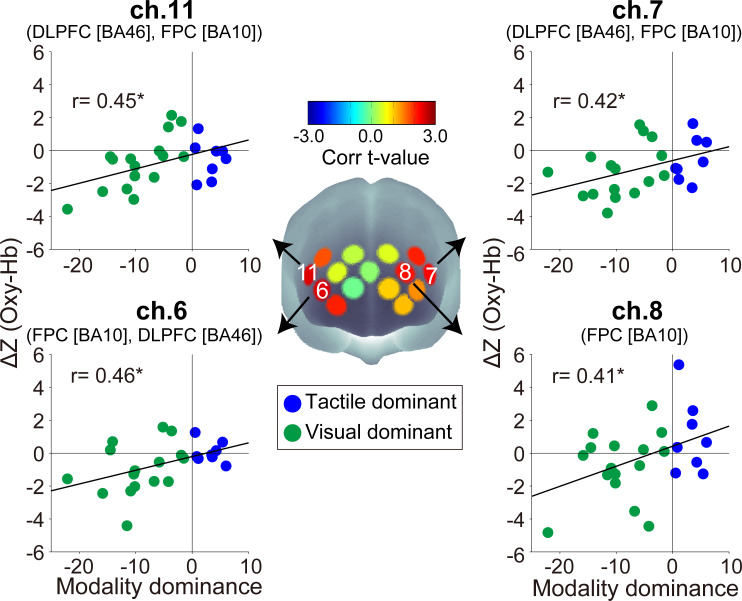
Spatial configurations of the t-values from correlation analysis between individual modality dominance and Δ*Z* (Oxy-Hb). Scatter plots show the inter-subject distributions in channels with significant correlation coefficients. The positive and negative values of Δ***Z*** indicate increasing and decreasing activity, respectively. BA: Brodmann area, *p < 0.05 (uncorrected).

We investigated whether there was a relationship between the inter-subject variance of WM modality dominance (i.e., performance in Task1) and that of fNIRS signal and found that there were significant correlations with neural activity changes of oxy-Hb in bilateral prefrontal areas (Δ*Z*) (left area: ch.7 r = 0.42, *p* = 0.039; ch.8 r = 0.41, *p* = 0.046; ch.12 r = 0.40, *p* = 0.0505. right area: ch.2 r = 0.40, *p* = 0.0502; ch.6 r = 0.46, *p* = 0.025; ch.11 r = 0.45, *p* = 0.026. other channels −0.023 < r < 0.36, *p* > 0.091) (Fig 3). Note that, among the channels with significant correlations between oxy-Hb Δ*Z* and WM modality dominance, only ch.7 also showed a significant correlation with the inter-subject variance of Δ*Freq* (ch.6: r = −0.23, *p* = 0.46; ch.7: r = −0.80, *p* = 0.0011; ch.8: r = −0.018, *p* = 0.95; ch.11: r = −0.31, *p* = 0.31). However, we found no evidence of a correlation between the inter-subject variance of oxy-Hb Δ*Z* and that of counting accuracy in the focused channels (ch.6: r = −0.22, *p* = 0.35; ch.7: r = −0.20, *p* = 0.41; ch.8: r = 0.12, *p* = 0.62; ch.11: r = −0.16, *p* = 0.50).

The inter-subject variance of prefrontal oxy-Hb activity strength itself (*Z*_*Init*_ and *Z*_*Final*_) was not significantly correlated with individual WM modality dominance quantified in the first searching task. Only the inter-subject oxy-Hb variance in *Z*_*Init*_ of ch.6 demonstrated a marginally significant correlation with WM modality dominance (ch.6: r = −0.40, *p* = 0.052; other channels: −0.32 < r < 0.19, *p*’s > 0.13). No channel showed a significant correlation (−0.026 < r < 0.35, *p*’s > 0.10) at the final activity level (*Z*_*Final*_). Regarding deoxy-Hb, the inter-subject variance of *Z*_*Init*_ and *Z*_*Final*_ were not significantly correlated with individual WM modality dominance (*Z*_*Init*_: −0.31 < r < 0.19, *p*’s > 0.14; *Z*_*Final*_: −0.17 < r < 0.23, *p*’s > 0.28). Only the inter-subject variance in Δ*Z* of ch.11 was significantly correlated with WM modality dominance (ch.11: r = 0.46, *p* = 0.024; other channels: −0.26 < r < 0.34, *p*’s > 0.11), but the deoxy-Hb Δ*Z* in ch.11 and Δ*Freq* did not show significant correlation (r = −0.13, *p* = 0.67).

**Fig 4:**
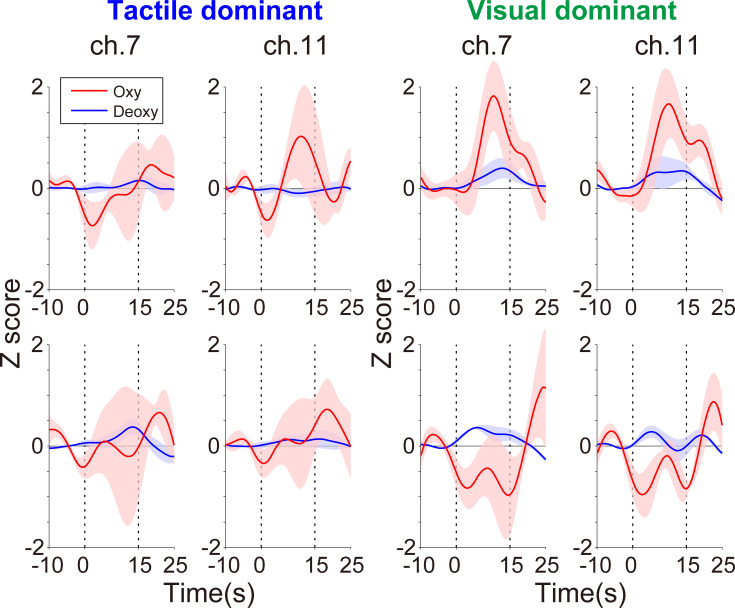
Temporal z scores in ch.7 [mainly left DLPFC (Brodmann area 46) and FPC (Brodmann area 10)] and ch.11 [right DLPFC (Brodmann area 46) and FPC (Brodmann area 10)]. The four left panels and four right panels show the average oxy-Hb (red) and deoxy-Hb (blue) responses in TD (participant nos. 1–5) and VD (participant nos. 20–24) individuals, respectively. The upper row shows the first two sessions and the lower row shows the last two sessions. The lighter colored regions around the time course lines indicate the standard error. Time zero in the horizontal axis indicates the start time of a task block.

In those channels with significant correlations between Δ*Z* of oxy-Hb and WM modality dominance as shown in Fig 3, individuals with stronger visual dominance showed decreasing activities during the cognitive-motor dual-task. As an example of the typical activity change in the current task, we present differences in the fNIRS signals of ch.7 and ch.11 between TD and VD individuals. Fig 4 shows the temporal z score profiles averaged across the task blocks during the 1st and 2nd sessions and the 7th and 8th sessions. Oxy-Hb profiles showed a marked difference between TD individuals (participant nos. 1–5) and VD individuals (participant nos. 20–24). TD individuals did not show large oxy-Hb changes over repeated fNIRS sessions. By contrast, oxy-Hb was reduced during later sessions in the VD individuals. No marked deoxy-Hb differences were observed between TD and VD individuals. The temporal profiles of the all recording channels are shown in S1 to S4 Figs in S1 File.

## Discussion

As shown in other cognitive functions such as motor imagery and focus of attention [9–11, 21], we found marked variation in individual WM ability characterized by qualitative dimensions based on the dominance of sensory modality. We also found that the prefrontal activities were significantly correlated with the degree of modality dominance, supporting our hypothesis that the DLPFC and FPC is one of the important regions encoding individual qualitative aspects of the WM system.

In addition to quantitative attributes, such as capacity [5, 6], individual WM ability has qualitative characteristics based on sensory modality dominance. The current findings of individual differences based on modality dominance are consistent with our previous studies that investigated attentional strategy and motor imagery [9–11]. This indicates that modality dominance for sensory processing, especially the contrast between tactile/somatosensory and visual processing, is a fundamental property common to extensive cognitive functions beyond WM. The mutual interactions between WM and attentional control [33–36] and models that propose attentional control as a critical aspect of WM executive function [37, 38] also support the importance of modality dominance as a qualitative aspect that characterizes individual cognitive functions.

Consistent with our previous studies [10, 21], we also confirmed that individual modality dominance is determined by the ability to process internal body information rather than the external environmental information such as visual stimuli. Indeed, in the current study, the inter-subject variance of modality dominance showed a significant correlation with searching cost only under the tactile condition (tactile condition: r = −0.62, *p* = 0.001. visual condition: r = 0.04, *p* = 0.87, see S5 Fig in S1 File), suggesting that qualitative WM ability could depend on whether individuals are accustomed to processing internal body information (rather than “which sensory modality is most efficiently encoded by WM”). The individuals with visual dominance may therefore rely on visual information because they are relatively poor at processing internal body information.

The current findings identified the bilateral DLPFC and FPC as one of the potential regions contributing to individual modality dominance in WM. Furthermore, the lack of correlation between the inter-subject variance of activity change in the bilateral DLPFC/FPC (i.e., Δ*Z* of oxy-Hb) and that of cognitive performance during fNIRS measurement supports the conclusion that individual WM ability rather than task difficulty is a stronger influential factor on the observed individual differences in bilateral DLFPC/FPC activities found in our cohort. Numerous studies have implicated DLPFC and FPC function in WM. For instance, the right prefrontal cortex showed significantly enhanced activity when individuals were required to maintain spatial information [16, 39]. The involvement of these areas for visuospatial WM was further suggested by a transcranial direct current stimulation study [15, 40] and a patient study [41]. On the other hand, while the left prefrontal cortex also contributes to maintaining visuospatial information in WM [16, 17], this area also processes internal body information such as tactile and somatosensory information during cognitive tasks [19, 20]. Hence, WM functions mediated by the right and left prefrontal areas could have distinct modality dependencies. However, bilateral prefrontal areas are strongly connected and this connectivity enhances cognitive and motor performance [42], implying that right and left prefrontal networks cooperatively process multimodal sensory information rather than independently processing different sensory modalities. Thus, the functional level of multimodal sensory processing in the right and left DLPFC/FPCs might confer modality dominance in WM. In the current study, the bilateral prefrontal area activities were reduced across repeated fNIRS sessions. Previous studies also reported a decrease in prefrontal activity associated with familiarity or higher skill level during cognitive tasks [43–45]. Thus, the decreased prefrontal activity observed in individuals with enhanced visual dominance may reflect efficient cognitive processing, especially the efficient multimodal sensory processing.

It is important to note that comparing bilateral prefrontal areas revealed that the right DLPFC/FPC is more likely to reflect individual modality dominance than the left DLPFC/FPC. There are two possible reasons for this finding. First, we cannot dissociate the effect of the motor component on left DLPFC/FPC activity. The left DLPFC/FPC is extensively connected to motor-related areas [46] and increased tapping frequency in the fNIRS task was associated with decreased neural activity in ch.7, the left DLPFC/FPC. Therefore, it is possible that reduced neural activity in the left prefrontal area represents a motor effect associated with the degree of motor automaticity. Second, individual modality dominance correlated with oxy-HB and deoxy-Hb signals in ch.11, the right DLPFC/FPC. Deoxy-Hb signals are less sensitive than oxy-Hb signals to changes in cerebral blood flow [47, 48]. Thus, the right DLPFC/FPC correlated with deoxy-Hb would have a higher sensitivity to the individual modality dominance. However, as shown in Fig 4, some deoxy-Hb profiles did not show classical responses such as an opposite response between oxy-Hb and deoxy-Hb (e.g., ch.7 and ch.11 in the first two sessions among the VD individuals). In the context of this unexpected finding, there is the possibility that the observed deoxy-Hb signal contains artifact components in addition to the neural activity reflecting individual WM ability. Therefore, we posit that the correlation observed in the deoxy-Hb signal may not be a critical one.

Based on the involvement of the prefrontal area in processing internal body information [13, 19, 20], we argue that differences in the prefrontal activities underly individual WM modality dominance and that modality dominance is a primary individual cognitive characteristic. However, we cannot fully reject the possibility that the prefrontal activities observed in the current study reflect individual perceptual sensitivity to internal body sensory inputs rather than cognitive processing. Nevertheless, it is certain that modality dominance conferred by the prefrontal cortex is a central qualitative aspect of the individual brain function.

Many recent studies have examined the potential for prefrontal area neuromodulation to improve cognitive or motor function, but the variability in neuromodulation training effects is high [49, 50]. This training variability among participants may be due to qualitative individual differences in brain function, as demonstrated in this study. Thus, the current findings will help to establish tailor-made intervention protocols to maximize individual training effects during neuromodulation procedures.

## Conclusions

We reveal qualitative individual differences in WM for specific sensory modalities. These differences were largely dependent on distinct prefrontal activity patterns, especially in the bilateral DLPFC and FPC. Thus, these regions are a critical neurological locus that mediates an individual’s modality dominance in cognitive function. Further, they might be an interventional target to develop personalized neuromodulation protocols in rehabilitation and sports training using transcranial direct current stimulation, transcranial magnetic stimulation, and neurofeedback.

## Supporting information

S1 File (PDF)

## References

[1] Baddeley A. Working memory. Curr Biol. 2010;20: R136–40. 10.1016/j.cub.2009.12.014 20178752

[2] Robertson EM, Tormos JM, Maeda F, Pascual-Leone A. The role of the dorsolateral prefrontal cortex during sequence learning is specific for spatial information. Cereb Cortex. 2001;11: 628–35. 10.1093/cercor/11.7.628 11415965

[3] Engle RW. Working memory capacity as executive attention. Curr Dir Psychol Sci. 2002;11: 19–23. 10.1111/1467-8721.00160

[4] Kane MJ, Bleckley MK, Conway AR, Engle RW. A controlled-attention view of working-memory capacity. J Exp Psychol Gen. 2001;130: 169–83. 10.1037//0096-3445.130.2.169 11409097

[5] Bo J, Seidler RD. Visuospatial working memory capacity predicts the organization of acquired explicit motor sequences. J Neurophysiol. 2009;101: 3116–25. 10.1152/jn.00006.2009 19357338 PMC2694099

[6] Unsworth N, Engle RW. Individual differences in working memory capacity and learning: evidence from the serial reaction time task. Mem Cognit. 2005;33: 213–20. 10.3758/bf03195310 16028576

[7] Vergeer I, Roberts J. Movement and stretching imagery during flexibility training. J Sports Sci. 2006;24: 197–208. 10.1080/02640410500131811 16368630

[8] Laessoe U, Hoeck HC, Simonsen O, Voigt M. Residual attentional capacity amongst young and elderly during dual and triple task walking. Hum Mov Sci. 2008;27: 496–512. 10.1016/j.humov.2007.12.001 18226839

[9] Sakurada T, Hirai M, Watanabe E. Optimization of a motor learning attention-directing strategy based on an individual’s motor imagery ability. Exp Brain Res. 2016;234: 301–311. 10.1007/s00221-015-4464-9 26466828

[10] Sakurada T, Nakajima T, Morita M, Hirai M, Watanabe E. Improved motor performance in patients with acute stroke using the optimal individual attentional strategy. Sci Rep. 2017;7: 40592 10.1038/srep40592 28094320 PMC5240116

[11] Sakurada T, Hirai M, Watanabe E. Individual optimal attentional strategy during implicit motor learning boosts frontoparietal neural processing efficiency: A functional near-infrared spectroscopy study. Brain Behav. 2019;9: e01183 10.1002/brb3.1183 30520270 PMC6346671

[12] Luck SJ, Vogel EK. Visual working memory capacity: from psychophysics and neurobiology to individual differences. Trends Cogn Sci. 2013;17: 391–400. 10.1016/j.tics.2013.06.006 23850263 PMC3729738

[13] Savini N, Brunetti M, Babiloni C, Ferretti A. Working memory of somatosensory stimuli: An fMRI study. Int J Psychophysiol. 2012;86: 220–228. 10.1016/j.ijpsycho.2012.09.007 23044088

[14] Engle RW, Tuholski SW, Laughlin JE, Conway ARA. Working memory, short-term memory, and general fluid intelligence: A latent-variable approach. J Exp Psychol Gen. 1999;128: 309–331. 10.1037//0096-3445.128.3.309 10513398

[15] Giglia G, Brighina F, Rizzo S, Puma A, Indovino S, Maccora S, et al Anodal transcranial direct current stimulation of the right dorsolateral prefrontal cortex enhances memory-guided responses in a visuospatial working memory task. Funct Neurol. 2014;29: 189–93. 10.11138/FNeur/2014.29.3.189 25473739 PMC4264786

[16] Slotnick SD, Moo LR. Prefrontal cortex hemispheric specialization for categorical and coordinate visual spatial memory. Neuropsychologia. 2006;44: 1560–1568. 10.1016/j.neuropsychologia.2006.01.018 16516248

[17] Owen AM, McMillan KM, Laird AR, Bullmore E. N-back working memory paradigm: a meta-analysis of normative functional neuroimaging studies. Hum Brain Mapp. 2005;25: 46–59. 10.1002/hbm.20131 15846822 PMC6871745

[18] Numminen J, Schürmann M, Hiltunen J, Joensuu R, Jousmäki V, Koskinen SK, et al Cortical activation during a spatiotemporal tactile comparison task. Neuroimage. 2004;22: 815–821. 10.1016/j.neuroimage.2004.02.011 15193610

[19] Pleger B, Ruff CC, Blankenburg F, Bestmann S, Wiech K, Stephan KE, et al Neural coding of tactile decisions in the human prefrontal cortex. J Neurosci. 2006;26: 12596–12601. 10.1523/JNEUROSCI.4275-06.2006 17135421 PMC2636906

[20] Kaas AL, Van Mier H, Goebel R. The neural correlates of human working memory for haptically explored object orientations. Cereb Cortex. 2007;17: 1637–1649. 10.1093/cercor/bhl074 16966490

[21] Sakurada T, Goto A, Tetsuka M, Nakajima T, Morita M, Yamamoto S-I, et al Prefrontal activity predicts individual differences in optimal attentional strategy for preventing motor performance decline: a functional near-infrared spectroscopy study. Neurophotonics. 2019;6: 025012 10.1117/1.NPh.6.2.025012 31259197 PMC6563944

[22] Oldfield RC. The assessment and analysis of handedness: The Edinburgh inventory. Neuropsychologia. 1971;9: 97–113. 10.1016/0028-3932(71)90067-4 5146491

[23] Funane T, Atsumori H, Katura T, Obata AN, Sato H, Tanikawa Y, et al Quantitative evaluation of deep and shallow tissue layers’ contribution to fNIRS signal using multi-distance optodes and independent component analysis. Neuroimage. 2014;85: 150–165. 10.1016/j.neuroimage.2013.02.026 23439443

[24] Akgül CB, Akin A, Sankur B. Extraction of cognitive activity-related waveforms from functional near-infrared spectroscopy signals. Med Biol Eng Comput. 2006;44: 945–58. 10.1007/s11517-006-0116-3 17061116

[25] Hirosaka R, Katura T, Kawaguchi H, Tanaka N, Iwamoto M. Noisy time-delayed decorrelation and its application to extraction of neural activity from single optical recordings in guinea pigs. Phys D Nonlinear Phenom. 2004;194: 320–332. 10.1016/J.PHYSD.2004.03.005

[26] Morren G, Wolf U, Lemmerling P, Wolf M, Choi JH, Gratton E, et al Detection of fast neuronal signals in the motor cortex from functional near infrared spectroscopy measurements using independent component analysis. Med Biol Eng Comput. 2004;42: 92–9. 10.1007/BF02351016 14977228

[27] Kohno S, Miyai I, Seiyama A, Oda I, Ishikawa A, Tsuneishi S, et al Removal of the skin blood flow artifact in functional near-infrared spectroscopic imaging data through independent component analysis. J Biomed Opt. 2007;12: 062111 10.1117/1.2814249 18163814

[28] Singh AK, Okamoto M, Dan H, Jurcak V, Dan I. Spatial registration of multichannel multi-subject fNIRS data to MNI space without MRI. Neuroimage. 2005;27: 842–851. 10.1016/j.neuroimage.2005.05.019 15979346

[29] Landers M, Wulf G, Wallmann H, Guadagnoli M. An external focus of attention attenuates balance impairment in patients with Parkinson’s disease who have a fall history. Physiotherapy. 2005;91: 152–158. 10.1016/j.physio.2004.11.010

[30] Wulf G, Landers M, Lewthwaite R, Töllner T. External focus instructions reduce postural instability in individuals with Parkinson disease. Phys Ther. 2009;89: 162–8. 10.2522/ptj.20080045 19074619

[31] Wulf G, Töllner T, Shea CH. Attentional focus effects as a function of task difficulty. Res Q Exerc Sport. 2007;78: 257–264. 10.1080/02701367.2007.10599423 17679499

[32] Matsuda G, Hiraki K. Sustained decrease in oxygenated hemoglobin during video games in the dorsal prefrontal cortex: A NIRS study of children. Neuroimage. 2006;29: 706–711. 10.1016/j.neuroimage.2005.08.019 16230030

[33] Furley P, Wood G. Working memory, attentional control, and expertise in sports: A review of current literature and directions for future research. J Appl Res Mem Cogn. 2016;5: 415–425. 10.1016/J.JARMAC.2016.05.001

[34] Wood G, Vine SJ, Wilson MR. Working memory capacity, controlled attention and aiming performance under pressure. Psychol Res. 2016;80: 510–7. 10.1007/s00426-015-0673-x 26021749

[35] Ducrocq E, Wilson M, Vine S, Derakshan N. Training attentional control improves cognitive and motor task performance. J Sport Exerc Psychol. 2016;38: 521–533. 10.1123/jsep.2016-0052 27736272

[36] Wang J-R, Hsieh S. Neurofeedback training improves attention and working memory performance. Clin Neurophysiol. 2013;124: 2406–2420. 10.1016/j.clinph.2013.05.020 23827814

[37] Miyake A, Friedman NP, Emerson MJ, Witzki AH, Howerter A, Wager TD. The unity and diversity of executive functions and their contributions to complex “Frontal Lobe” tasks: a latent variable analysis. Cogn Psychol. 2000;41: 49–100. 10.1006/cogp.1999.0734 10945922

[38] Unsworth N, Redick TS, Spillers GJ, Brewer GA. Variation in working memory capacity and cognitive control: goal maintenance and microadjustments of control. Q J Exp Psychol. 2012;65: 326–55. 10.1080/17470218.2011.597865 21851149

[39] Owen AM, Evans AC, Petrides M. Evidence for a two-stage model of spatial working memory processing within the lateral frontal cortex: a positron emission tomography study. Cereb cortex. 1996;6: 31–8. 10.1093/cercor/6.1.31 8670636

[40] Wu Y-J, Tseng P, Chang C-F, Pai M-C, Hsu K-S, Lin C-C, et al Modulating the interference effect on spatial working memory by applying transcranial direct current stimulation over the right dorsolateral prefrontal cortex. Brain Cogn. 2014;91: 87–94. 10.1016/j.bandc.2014.09.002 25265321

[41] van Asselen M, Kessels RPC, Neggers SFW, Kappelle LJ, Frijns CJM, Postma A. Brain areas involved in spatial working memory. Neuropsychologia. 2006;44: 1185–1194. 10.1016/j.neuropsychologia.2005.10.005 16300806

[42] Lin C-HJ, Chiang M-C, Wu AD, Iacoboni M, Udompholkul P, Yazdanshenas O, et al Enhanced motor learning in older adults is accompanied by increased bilateral frontal and fronto-parietal connectivity. Brain Connect. 2012;2: 56–68. 10.1089/brain.2011.0059 22512355

[43] Jansma JM, Ramsey NF, Slagter HA, Kahn RS. Functional anatomical correlates of controlled and automatic processing. J Cogn Neurosci. 2001;13: 730–43. 10.1162/08989290152541403 11564318

[44] Ramsey NF, Jansma JM, Jager G, Van Raalten T, Kahn RS. Neurophysiological factors in human information processing capacity. Brain. 2004;127: 517–525. 10.1093/brain/awh060 14691061

[45] Koike S, Takizawa R, Nishimura Y, Kinou M, Kawasaki S, Kasai K. Reduced but broader prefrontal activity in patients with schizophrenia during n-back working memory tasks: a multi-channel near-infrared spectroscopy study. J Psychiatr Res. 2013;47: 1240–6. 10.1016/j.jpsychires.2013.05.009 23743135

[46] Kim YK, Park E, Lee A, Im C-H, Kim Y-H. Changes in network connectivity during motor imagery and execution. He B, editor. PLoS One. 2018;13: e0190715 10.1371/journal.pone.0190715 29324886 PMC5764263

[47] Hoshi Y. Functional near-infrared optical imaging: utility and limitations in human brain mapping. Psychophysiology. 2003;40: 511–20. 10.1111/1469-8986.00053 14570159

[48] Strangman G, Culver JP, Thompson JH, Boas DA. A quantitative comparison of simultaneous BOLD fMRI and NIRS recordings during functional brain activation. Neuroimage. 2002;17: 719–731. 10.1016/S1053-8119(02)91227-9 12377147

[49] Alkoby O, Abu-Rmileh A, Shriki O, Todder D. Can we predict who will respond to neurofeedback? A review of the inefficacy problem and existing predictors for successful EEG neurofeedback learning. Neuroscience. 2018;378: 155–164. 10.1016/j.neuroscience.2016.12.050 28069531

[50] Emmert K, Kopel R, Sulzer J, Brühl AB, Berman BD, Linden DEJ, et al Meta-analysis of real-time fMRI neurofeedback studies using individual participant data: How is brain regulation mediated? Neuroimage. 2016;124: 806–812. 10.1016/j.neuroimage.2015.09.042 26419389

