# Supplementary material for: Distinct bilateral prefrontal activity patterns associated with the qualitative aspect of working memory characterized by individual sensory modality dominance
Source: PLoS One. 2020 Aug 26;15(8):e0238235. doi: 10.1371/journal.pone.0238235 (PMC7449398; doi:10.1371/journal.pone.0238235)
Supplement: S1 File — (PDF) [file pone.0238235.s001.pdf]

Supporting Information

Title

Distinct bilateral prefrontal activity patterns associated with the qualitative aspect of working memory characterized by individual sensory modality dominance

Authors
Mayuko Matsumoto, Takeshi Sakurada and Shin-ichiroh Yamamoto

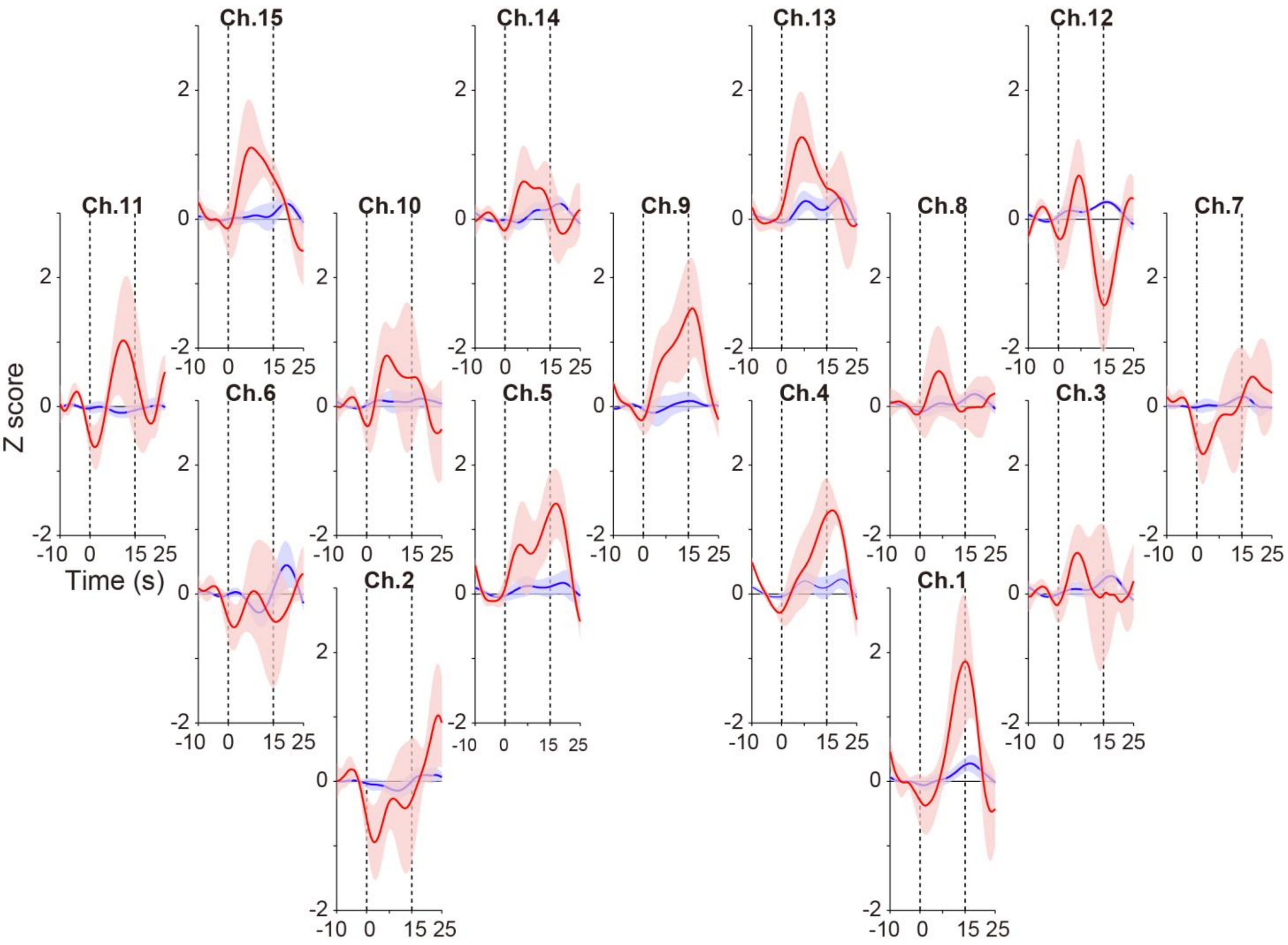

S1 Fig. Typical temporal profiles of all recording channels in the TD individuals (first 2 sessions).

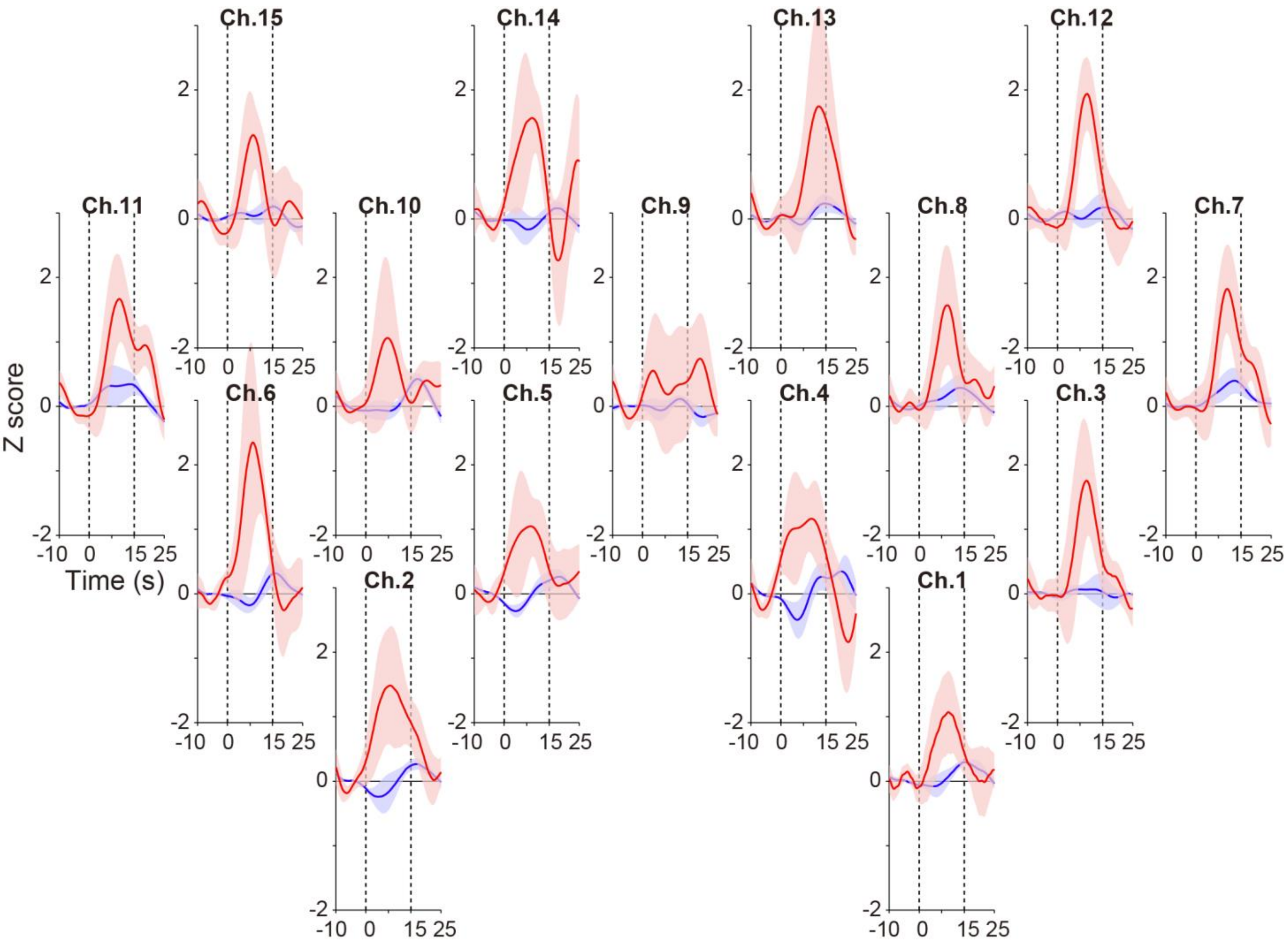

S2 Fig. Typical temporal profiles of all recording channels in the VD individuals (first 2 sessions).

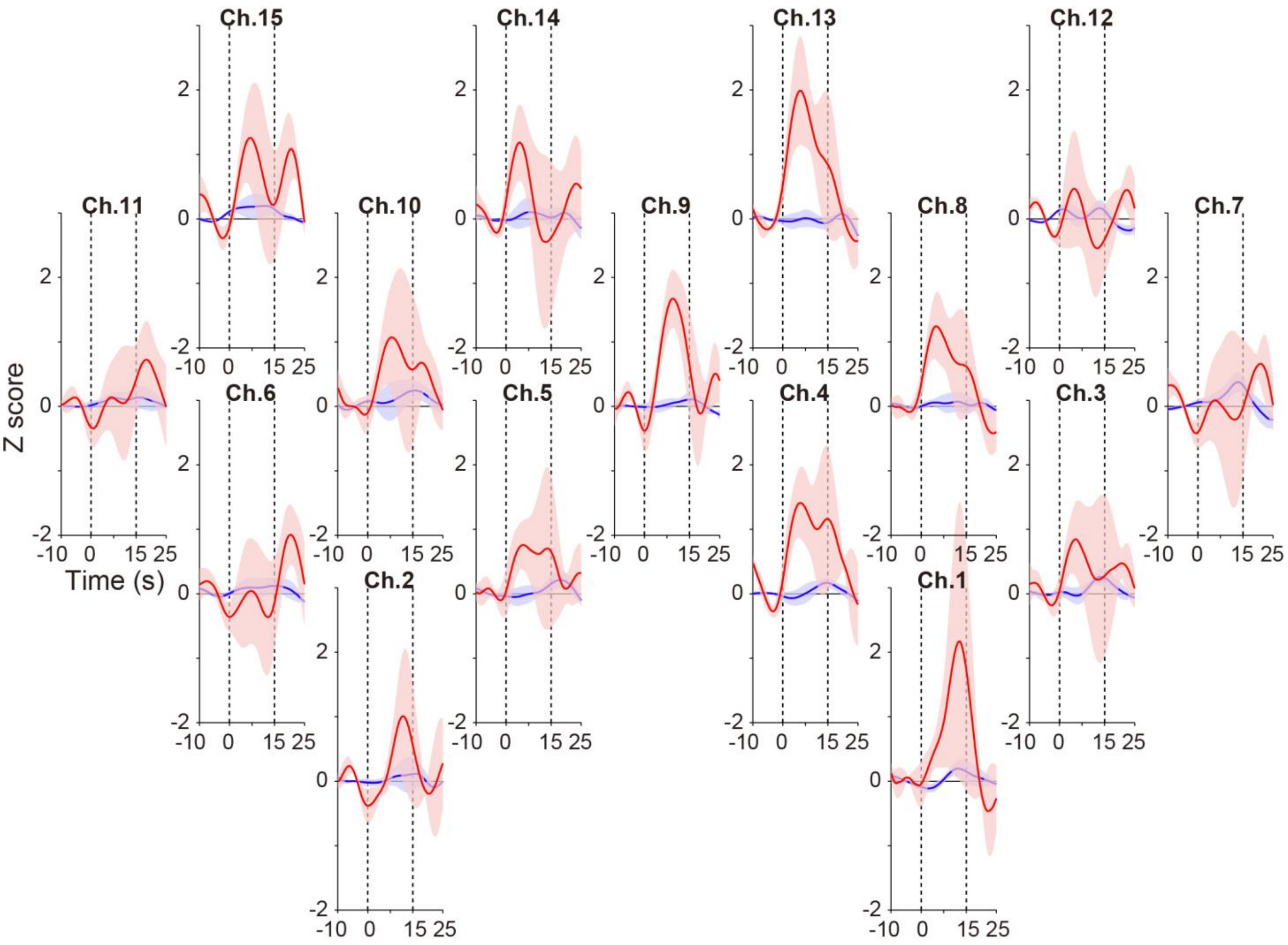

S3 Fig. Typical temporal profiles of all recording channels in the TD individuals (last 2 sessions).

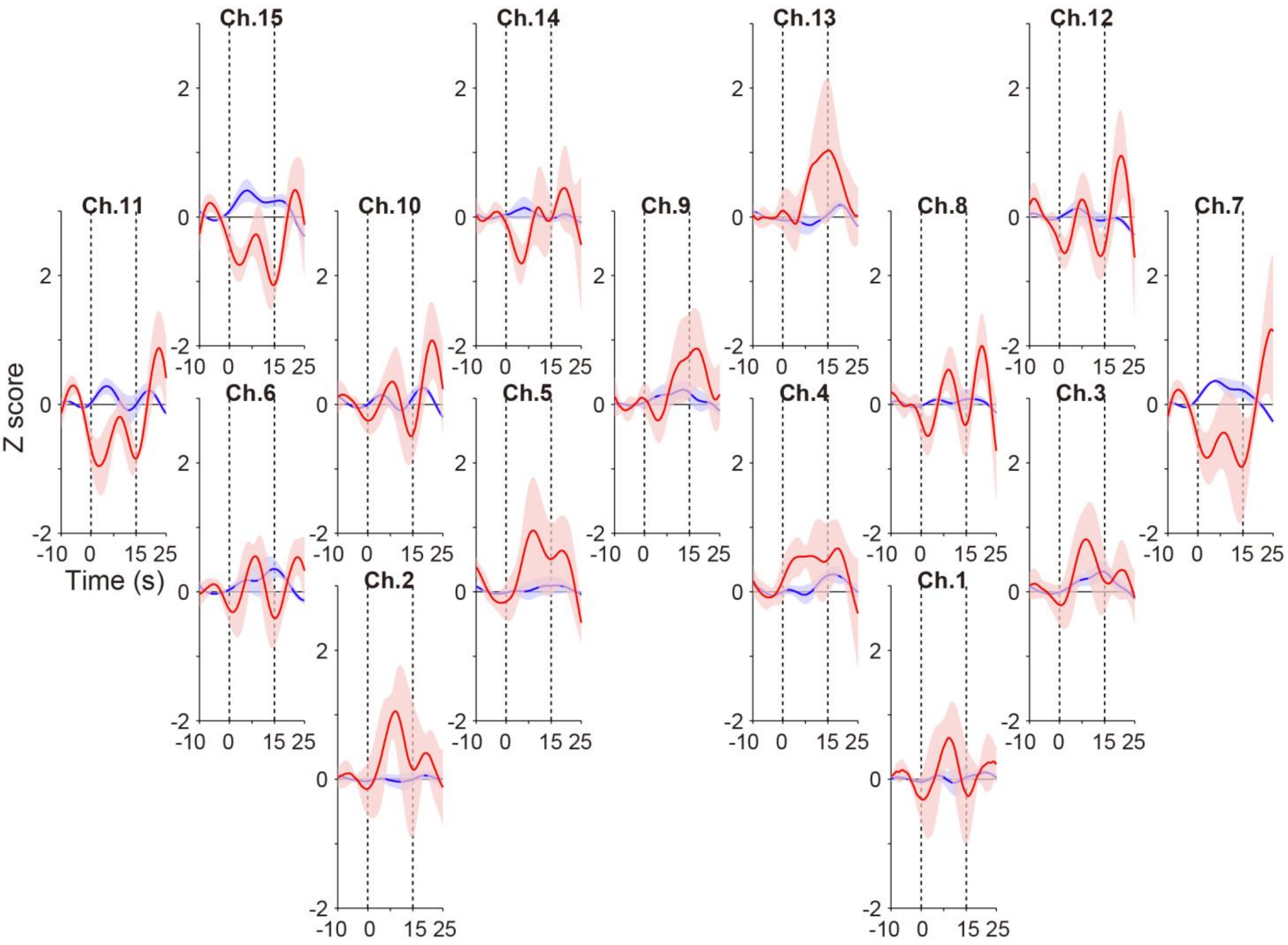

S4 Fig. Typical temporal profiles of all recording channels in the VD individuals (last 2 sessions).

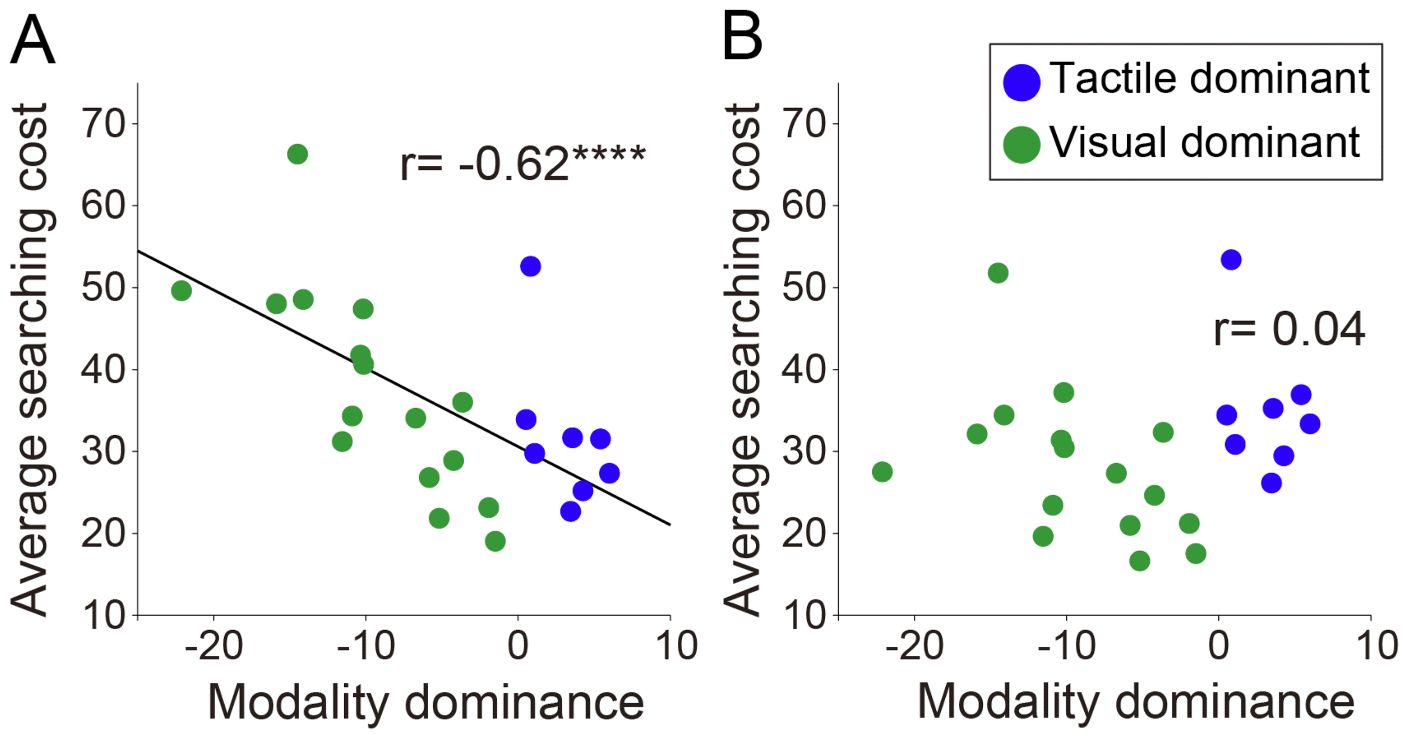

S5 Fig. Correlations between modality dominance and searching cost in the tactile (A) and visual (B) conditions.

The horizontal axis indicates the individual modality dominance and the longitudinal axis indicates the individual mean searching cost averaged from the 2nd to 15th trials. ****$p < 0.001$
